# [Retracted] MicroRNA‑143 inhibits cell migration and invasion by targeting matrix metalloproteinase 13 in prostate cancer

**DOI:** 10.3892/mmr.2023.12944

**Published:** 2023-01-19

**Authors:** Deyao Wu, Ping Huang, Linmao Wang, Yunfeng Zhou, Huixing Pan, Ping Qu

Mol Med Rep 8: 626–630, 2013; DOI: 10.3892/mmr.2013.1501

Following the publication of this paper, it was drawn to the Editor's attention by a concerned reader that several of the panels showing the results from cell migration and invasion assay experiments in Fig. 2 on p. 628 contained sections of data that were overlapping with other panels within the same figure.

Given the issue of the overlapping sections of data, the Editor of *Molecular Medicine Reports* has decided that this paper should be retracted from the Journal on account of an overall lack of confidence in the presented results. The authors were asked for an explanation to account for these concerns, but the Editorial Office did not receive a satisfactory reply. The Editor apologizes to the readership for any inconvenience caused.

